# Intra-articular temperature monitoring during radiofrequency ablation in ex-vivo bovine hip joints via Fiber Bragg grating sensors

**DOI:** 10.1186/s12891-023-06836-6

**Published:** 2023-09-28

**Authors:** Umile Giuseppe Longo, Francesca De Tommasi, Giuseppe Salvatore, Alberto Lalli, Daniela Lo Presti, Carlo Massaroni, Emiliano Schena

**Affiliations:** 1 Fondazione Policlinico Universitario Campus Bio-Medico, Via Alvaro del Portillo, 200, Roma, 00128 Italy; 2 Research Unit of Orthopaedic and Trauma Surgery, Department of Medicine and Surgery, Università Campus Bio-Medico di Roma, Via Alvaro del Portillo, 21, Roma, 00128 Italy; 3 Unit of Measurements and Biomedical Instrumentation, Università Campus Bio-Medico di Roma, via Alvaro del Portillo, 200, Trigoria, Rome, 00128 Italy

**Keywords:** Radiofrequency ablation, Fiber optic sensors, Fiber Bragg grating sensors, Joint temperature, Hip arthroscopy

## Abstract

**Purpose:**

Radiofrequency ablation is an increasingly used surgical option for ablation, resection and coagulation of soft tissues in joint arthroscopy. One of the major issues of thermal ablation is the temperature monitoring across the target areas, as cellular mortality is a direct consequence of thermal dosimetry. Temperatures from 45 °C to 50 °C are at risk of damage to chondrocytes. One of the most reliable tools for temperature monitoring is represented by fiber optic sensors, as they allow accurate and real-time temperature measurement via a minimally invasive approach. The aim of this study was to determine, by fiber Bragg grating sensors (FBGs), the safety of radiofrequency ablation in tissue heating applied to ex-vivo bovine hip joints.

**Methods:**

Ex vivo bovine hips were subjected to radiofrequency ablation, specifically in the acetabular labrum, for a total of two experiments. The WEREWOLF System (Smith + Nephew, Watford, UK) was employed in high operating mode and in a controlled ablation way. One optical fiber embedding seven FBGs was used to record multipoint temperature variations. Each sensor was 1 mm in length with a distance from edge to edge with each other of 2 mm.

**Results:**

The maximum variation was recorded in both the tests by the FBG1 (i.e., the closest one to the electrode tip) and was lower than to 2.8 °C. The other sensors (from FBG2 to FBG7) did not record a significant temperature change throughout the duration of the experiment (maximum up to 0.7 °C for FBG7).

**Conclusions:**

No significant increase in temperature was observed at any of the seven sites. The sensor nearest to the radiofrequency source exhibited the highest temperature rise, but the variation was only 3 °C. The minimal temperature increase registered at the measurement sites, according to existing literature, is not expected to be cytotoxic. FBGs demonstrate the potential to fulfil the strict requirements for temperature measurements during arthroscopic surgery.

## Introduction

Radiofrequency ablation in arthroscopy is an increasingly used surgical option for ablation, resection, and coagulation of soft tissues [4, 24, 36]. The development of the radiofrequency technique was applied in orthopedics to decrease laxity of connective tissues around joints, at first in the context of shoulder instability. The rationale behind it was to obtain temperatures between 70 °C and 80 °C, which would cause collagen to shrink and trigger a healing response [2, 12, 15, 21, 33]. This method involves inserting electrodes into a target site to generate an electrical current, causing frictional agitation at the ionic level and a consequent heat generation [1, 26].

However, radiofrequency energy also presents some hazards, as well as time-dependent consequences that clinicians must consider [28, 36].

One of the main challenges of thermal ablation is the measurement of temperature across the target sites, because cellular mortality is a direct consequence of thermal dosimetry. This task is challenging as all thermal procedures induce heat patterns in the tissue with high spatial and temporal gradients, frequently exceeding 50 °C/mm and 5 °C/s [4, 32, 35].

Several publications reported the risk of complications following thermal ablation during arthroscopic procedures. These devices raise the temperature of joint fluid, potentially leading to skin burns, nerve lesions, capsular damage and extensive chondrolysis. These complications may be the result of an excessive transmission of heat in the nearby tissues [7, 13, 14, 24, 25, 27].

The risk for chondrocyte damage is particularly relevant when it comes to the hip joint. In the hip’s central compartment, factors predisposing to chondrocyte damage comprise insufficient saline volume, extensive cartilage coverage, risk for poor saline flow, and direct proximity of weight-bearing cartilage to the site where the radiofrequency is applied [24].

Chondrocytes are damaged at temperatures ranging from 45 °C to 50 °C and damage arises after an elevation of only 8 °C [5, 24, 34].

For these reasons, temperature monitoring during radiofrequency ablation treatments may become a key aspect of minimally invasive procedures in arthroscopic surgeries, since it allows the surgeon to adjust temperature distribution in the tissue proximal to the radiofrequency electrodes and it diminishes the risk of potentially adverse effects to the nearby healthy tissue [26].

The use of thermocouples is the standard procedure for in-situ temperature measurement. These sensors, however, present two significant weaknesses: thermocouples may interfere with heating propagation since they consist of two metallic wires, and they are only able to measure the temperature in a single point, while they are unable to define a spatial profile of temperature [22, 31].

An alternative to thermocouples is thermal imaging, which exploits magnetic resonance imaging (MRI) or computed tomography (CT). However, these techniques also come with major limitations in terms of costs, exposure to X-rays and presence of artefacts [4, 9, 30].

Fiber optic sensors constitute the primary alternative to these techniques and have been recently applied for real-time temperature detection during radiofrequency ablation. Most importantly, specific fiber optic sensors (i.e., fiber Bragg grating sensors, FBGs) can outperform thermocouples due to their unique property of detecting temperature patterns on a single fiber with resolutions ranging from 0.1 to 10 mm, and the potential to multiplex across multiple fibers to measure the temperature pattern in one- or two-dimensional geometries [17].

Given the challenges posed by obtaining instantaneous and precise temperature data via a minimally invasive approach, multi-point temperature measurements obtained via FBGs appear to be a promising answer to these challenges [9].

Currently, some studies have tried to assess the variations in joint temperature during arthroscopic procedures on the shoulder and knee, both in human and animal models [2, 7, 10, 23, 24]. However, there is still a lack of evidence determining variations in joint and tissue temperature during radiofrequency ablation treatments in the hip joint. Moreover, none of the available studies used FBG technology.

The aim of the current study was to evaluate, by FBGs, the safety of radiofrequency ablation in tissue heating applied to ex-vivo bovine hip joints.

## Materials and methods

Ex vivo bovine hips were subjected to radiofrequency ablation during hip arthroscopy, specifically in the acetabular labrum. The WEREWOLF System (Smith + Nephew, Watford, UK) was employed in high operating mode and in a controlled ablation way (also referred as coablation). No limits to the temperature sensor were set, in order to assess the maximal excursion that is reached in the presented settings and decrease potential bias in the experiment. The joint perfusion pump used during hip arthroscopy was set at 50 mmHg. The water temperature used during hip arthroscopy typically ranges from 20 °C to 25 °C.

The co-ablation was activated with no arthroscopic manipulation. Aspiration via the probe was connected to the operating room aspiration system all the time and at maximum intensity. This modality allows the achievement of lower temperatures with respect to the traditional radiofrequency ablation method, thus reducing the risk of energy dissipation in the neighboring structures. This technology involves a generator through which the desired settings can be set and an electrode responsible for conveying the energy to the tissue. The tests were executed for around 20 s at room temperature (i.e., 25 °C), assessed through a thermocouple proximally to the joint surface before starting.

Standard hip arthroscopy was performed in a adult bovine hips.

**Fig. 1:**
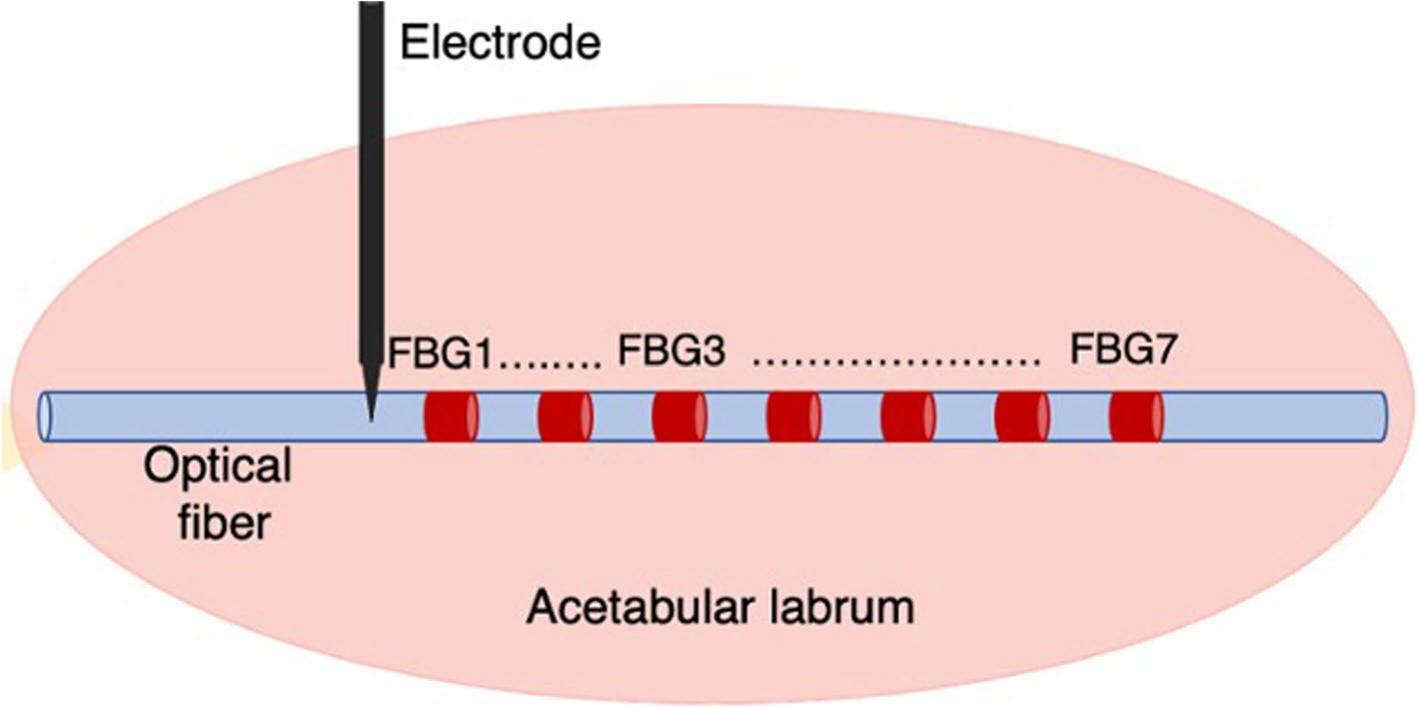
FBGs and electrode positioning in the acetabular labrum

 The optical fiber was positioned in the acetabular labrum by means of an 18 gauge biopsy needle and in the proximity of the RF electrode. The optical fiber embedding seven FBGs (FBG1 closer to the electrode tip and the others accordingly to Fig. 1) from AtGrating Technologies was used to record temperature variations in the acetabular labrum. This solution based on multiple sensors allowed us to perform multi-point measurements (in 7 sites) within the acetabular labrum. Each FBG was 1 mm in length with a distance from edge to edge with each other of 2 mm. They were characterized by reflectivity values around 65% and λ_B_ ones ranging from 1512 to 1588 nm.

Basically, an FBG consists of an optical fiber portion inscribed in its core. The so-called grating represents a periodic perturbation of the refractive index of the fiber core. So, when a broadband light spectrum is transmitted inside the fiber, a narrow spectrum is reflected backward in correspondence with the grating. Such retroreflected signal is centered around the Bragg wavelength (${{\uplambda }}_{\text{B}}$), satisfying the following Eq. [1] :

1 $${\lambda }_{B}= 2\cdot {n}_{eff}\cdot {\Lambda }$$

where ${n}_{eff}$ is the effective refractive index and ${\Lambda }$ is the grating period. When a fiber is exposed to strain ($\epsilon$) or temperature variation ($\varDelta \text{T}$), a Bragg wavelength shift (${\varDelta {\uplambda }}_{\text{B}}$) occurs. In this study, FBGs was used as temperature sensors only considering a strain-free configuration. Thus, by tracking ${\varDelta {\uplambda }}_{\text{B}}$ over time, $\varDelta \text{T}$ can be derived using the following relationship:

2 $${\varDelta {\uplambda }}_{\text{B}}= \text{S}\text{T}\cdot \varDelta \text{T}$$

where S_T_ is the thermal sensitivity (i.e., 10 pm$\cdot$C^−1^ in the case of the sensors used in this study). During the experiments, FBGs’ response was collected by means of an optical interrogator (si255 based on Hyperion Platform, Micron Optics, Atlanta, GA, USA) at a sampling frequency of 100 Hz.

## Results

**Fig. 2:**
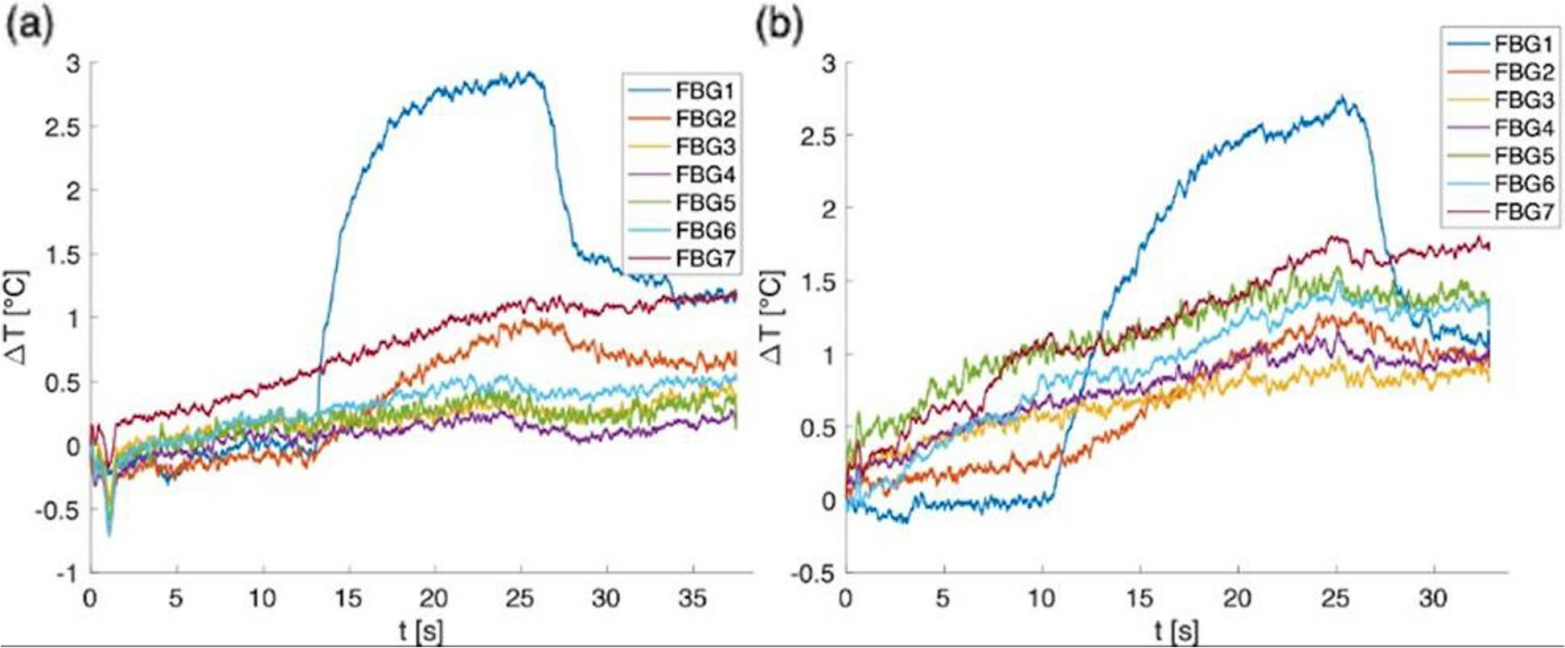
Temperature variations collected during the first (**a**) and the second (**b**) RF experiments carried out with the WEREWOLF System and measured by the seven FBGs of the optical fiber employed

 After experiments, FBGs data were examined in MATLAB (Mathworks, Natick, MA, USA) environment to retrieve the temperature trends recorded during the RF discharge. Figure 2a and b report the results obtained for the two experiments carried out with the WEREWOLF System. As shown, in this case, maximum $\varDelta \text{T}$ was recorded in both cases by the FBG1 (i.e., the one close to the electrode tip) and was lower than to 2.8 °C. The other sensors (from FBG2 to FBG7) did not record a significant temperature change throughout the duration of the experiment (maximum up to 0.7 °C for FBG7).

## Discussion

Radiofrequency ablation is increasingly used for soft tissue ablation, resection, and coagulation in arthroscopy. However, it presents hazards and time-dependent consequences that clinicians must consider. Precise temperature measurement is crucial to avoid complications and tissue damage. FBGs offer a promising alternative to standard thermocouples and thermal imaging due to their ability to detect temperature patterns on a single fiber with high resolutions. While some studies have explored joint temperature variations during arthroscopic procedures, there is a lack of evidence for hip joint radiofrequency ablation, especially using FBGs. This study aimed to assess the safety of radiofrequency ablation in tissue heating applied to ex-vivo bovine hip joints using FBGs.

The main finding of this study is that the slight temperature increase detected at the measurement sites, based on the literature data, should not be cytotoxic [5, 24, 34].

The sensors covered a surface area ranging from 3 to 22 mm from the radiofrequency source. The sensor closest to the radiofrequency source resulted in measuring the highest temperature increase. Nonetheless, the measured increase was of only three degrees, and, therefore, not cytotoxic for the surrounding tissue.

The focus of the current study on the hip joint is of significant relevance, given hip-specific characteristics that warrant consideration. The significant reduction in joint space and the absence of fluid-heating capacity buffer in the hip could potentially amplify the increase in temperature caused by the application of radiofrequencies, making the hip joint more susceptible to thermal damage [24, 29, 37].

McKormick et al. had previously investigated the role of joint fluid lavage in preserving safe temperatures (< 50°) in the hip joint and showed that the constant application of radiofrequency for 90 s at the capsulolabral junction can considerably increase intra-articular temperatures near (1 and 2 mm) and far (5 and 1 mm) from the radiofrequency source to values linked with irreversible chondrocyte toxicity [24].

Recent interests have been poured in newer devices that exploit a plasma bubble that allows for more effective debridement and decreased heat transmission to the joint fluid and nearby tissue. However, their benefits over standard radiofrequency protocols are yet to be verified. Faruque et al. in their randomized control trial compared intra-articular temperature profile in standard ablation versus plasma ablation radiofrequency tools for rotator cuff repair. 17.5% of their patients showed temperatures above 45 °C but no significant variations where observed between standard and plasma radiofrequency devices [10].

Moreover, the safety of both bipolar and coablation systems in contexts of subacromial decompression, rotator cuff surgery and impingement syndrome has been demonstrated, showing similar results for both devices [7, 8, 11, 16].

For these reasons, it is important to consider all the possible variables that may influence intra-articular temperature during arthroscopic procedures. According to Zoric et al., the three primary factors that affect the quantity and effect of heat produced during arthroscopic surgery are: the flow rate of the irrigation fluid, the duration of application of radiofrequency ablation, and the distance between the probe tip and target tissue [23]. The most significant effect came from the flow, and it was crucial to understand that in situations when the flow was restricted or stopped entirely, temperatures might exceed 50 °C after only 5 s of ablation [38].

On the other hand, the temperature of the irrigation fluid has been proposed as the most influential variable in intra-articular temperature variation during arthroscopic procedures [3, 6, 7].

Regarding knee joint arthroscopy, no difference were found in temperature generation between standard radiofrequency ablation and plasma ablation [23].

The current study has several points of strength. To the author’s knowledge, this is the first study assessing intra-articular temperature variations upon radiofrequency treatments in the hip joint. Also, FBGs were exploited for temperature sensing and, given their unique property of detecting temperature patterns on a single fiber with resolutions ranging from 0.1 to 10 mm, and the potential to multiplex across several fibers to catch the temperature pattern in one- or two-dimensional geometries, they can be considered among the most reliable tools for temperature detection within arthroscopic procedures.

The present article also has some limitations. First, the findings of this study are limited to the hip joint and cannot be extrapolated to other joints due to the peculiar characteristics of the joint in question. The authors are fully aware of the fact that the volume of the cow’s hip joint is not directly comparable to that of a human. One of our future objectives will be to replicate similar experiments in different joints, both in ex vivo and in vivo models. The volume of the joint can indeed influence the temperature of the fluid and co-ablation.

Moreover, the radiofrequency devices were tested using ex-vivo bovine tissues and, despite the fact that these modalities represent reasonable surrogates, the characteristics of these environments differ from the environment of real, human intra-operative procedures [19, 20].

In this study, ex vivo bovine hips were utilized for radiofrequency ablation procedures targeted at the acetabular labrum during hip arthroscopy. It is important to note that a ex vivo bovine hip lacks both circulation and body temperature, which significantly differs from the conditions present within a living organism. Despite maintaining a constant room temperature, the dynamics of water temperature in an ex vivo setting are expected to diverge from those observed in a living body. Therefore, a future endeavor will be to conduct future experiments in an in vivo setting, where at least some circulatory dynamics are present to better simulate real-life conditions.

On the other hand, to our knowledge, this is the first radiofrequency ablation study using the fiber bragg grating sensors, providing preliminary results of feasibility in this rapidly evolving and highly relevant research field.

Additionally, the intrinsic anatomical differences in soft tissue thickness between human and bovine models represent a limitation of the current article. The aim of the current study was to determine the feasibility of the exploited system in a cadaveric model, not to determine a specific temperature threshold after which cytotoxic damage would occur.

Additionally, the reported findings are limited to coablation radiofrequency systems, and do not consider temperature variations that may be caused by standard radiofrequency devices, although similar outcomes have been reported among the different systems [18].

The technology used in this pilot study is promising as testified by the broad acceptance in other medical and bioengineering applications. It can represent the future gold standard to evaluate the temperature increase in nearby tissue during radiofrequency treatments.

Given the growing use of arthroscopy all over the world and consequently of radiofrequencies, it will be important to evaluate the cytotoxicity values ​​for each of the tissues around the joints (tendons, ligaments, muscles, cartilages, bones). FBGs may fulfil the strict requirements for temperature measurements during arthroscopic surgery.

## Conclusions

No significant increase in temperature was observed at any of the seven sites. The sensor nearest to the radiofrequency source exhibited the highest temperature rise, but the variation was only 3 °C. The minimal temperature increase registered at the measurement sites, according to existing literature, is not expected to be cytotoxic. FBGs demonstrate the potential to fulfil the strict requirements for temperature measurements during arthroscopic surgery.

## Data Availability

The data presented in this study are available on request from the corresponding author.
